# Contrasting Electroencephalography-Derived Entropy and Neural Oscillations With Highly Skilled Meditators

**DOI:** 10.3389/fnhum.2021.628417

**Published:** 2021-04-30

**Authors:** Jacob H. Young, Martha E. Arterberry, Joshua P. Martin

**Affiliations:** ^1^Department of Biology, Colby College, Waterville, ME, United States; ^2^Department of Psychology, Colby College, Waterville, ME, United States

**Keywords:** meditation, electroencephalography, oscillations, power spectra, entropy, Lempel–Ziv

## Abstract

Meditation is an umbrella term for a number of mental training practices designed to improve the monitoring and regulation of attention and emotion. Some forms of meditation are now being used for clinical intervention. To accompany the increased clinical interest in meditation, research investigating the neural basis of these practices is needed. A central hypothesis of contemplative neuroscience is that meditative states, which are unique on a phenomenological level, differ on a neurophysiological level. To identify the electrophysiological correlates of meditation practice, the electrical brain activity of highly skilled meditators engaging in one of six meditation styles (shamatha, vipassana, zazen, dzogchen, tonglen, and visualization) was recorded. A mind-wandering task served as a control. Lempel–Ziv complexity showed differences in nonlinear brain dynamics (entropy) during meditation compared with mind wandering, suggesting that meditation, regardless of practice, affects neural complexity. In contrast, there were no differences in power spectra at six different frequency bands, likely due to the fact that participants engaged in different meditation practices. Finally, exploratory analyses suggest neurological differences among meditation practices. These findings highlight the importance of studying the electroencephalography (EEG) correlates of different meditative practices.

## Introduction

Meditation is a catch-all term referring to a diverse collection of mental exercises (Cahn and Polich, 2006; Fox et al., 2016). Generally, meditation practices involve the intentional monitoring and regulation of attention and emotion, which may be improved with regular practice (Lutz et al., 2008, 2009; Slagter et al., 2011; Tang et al., 2015). Meditation practices are now being effectively employed in a number of therapeutic domains (Rubia, 2009; Vøllestad et al., 2012, Simkin and Black, 2014; Eisendrath, 2016).

In recent years, there has been an increase in studies examining the neural basis for these practices (Van Dam et al., 2018). Scientific interest in meditation grew in the late seventies, with researchers examining the psychological and cognitive correlates, creating theories, proposing clinical applications, and beginning neurological study (Andresen, 2000). The earliest studies exploring the neurological correlates of meditation were conducted in Asia with advanced yogic meditators in India and Zen meditators in Japan using electroencephalography (EEG; Lutz et al., 2007).

Recently, there has been a movement within the field of contemplative neuroscience to address the methodological and theoretical issues facing the field (Cahn and Polich, 2006; Tang, 2012; Thomas and Cohen, 2014; Davidson and Kaszniak, 2015). However, few studies have made direct comparisons between the neurological correlates of different meditation practices. Instead, they almost exclusively studied one technique (Lutz et al., 2004; Hölzel et al., 2011; Nolfe, 2012; Davidson and Kaszniak, 2015; Fox et al., 2016). Emerging research suggests that meditation practices that differ on a psychological level also differ on a neurophysiological scale (Lutz et al., 2008; Travis and Shear, 2010; Tomasino et al., 2013; Fox et al., 2014, 2016; Lomas et al., 2015). However, most comparative research on meditation has been conducted through meta-analysis. This method is limited because it compares studies with different methodologies, inclusion criteria, and measurement tools. Additionally, a meta-analysis of many small studies does not necessarily predict the results of a single large study (Slavin, 1986). Furthermore, meta-analysis allows researchers to cherry-pick studies and disregard others, where possible (Stegenga, 2011). Therefore, an experiment designed to differentiate multiple styles with the same procedure would allow for a direct exploration of the potential neurological signatures of different practices.

Despite the methodological issues in research investigating the neurological correlates of meditation, preliminary findings are already being used in products marketed toward consumers. Some companies are marketing neurofeedback devices for EEG-assisted meditation. Proponents of EEG-assisted meditation assert that, if reproducible, EEG markers can be linked to specific meditation practices; learning to generate similar signals could aid in meditation practice (Brandmeyer and Delorme, 2013). However, there have been no large-scale studies investigating the neurological correlates of meditation styles. Moreover, the increase in EEG-assisted meditation is concerning because neurofeedback has been shown to have adverse effects when used improperly (Hammond and Kirk, 2008).

While meditation, in general, has become the object of increased scientific attention, this work is limited to a small number of meditation practices while ignoring others (Lutz et al., 2007; Fox et al., 2014; Matko and Sedlmeier, 2019). Practices that involve visualization, compassion, and non-dual awareness are rarely studied (Josipovic, 2014). The issues caused by the lack of study and differentiation of disparate meditation practices are not limited to scientific inquiry. Popular press articles also tout the benefits of “meditation” without providing specifics on what type of practices were used.

Meditation is an umbrella term encompassing a large number of distinct mental exercises, which differ in phenomenological character (Hölzel et al., 2011; Vago and David, 2012; Nash and Newberg, 2013; Dahl et al., 2015; Matko and Sedlmeier, 2019). A concrete definition of meditation remains elusive because of the vast number of cognitive processes it describes. For example, reciting a word or phrase (mantra meditation), the progressive relaxation of muscles (relaxation response), paying attention to a specific object (concentration), paying attention to many aspects of the present moment nonjudgmentally (mindfulness), and movement-based practices (yoga, tai chi, and qi cong) are all considered meditation in the scientific literature. Additionally, some of these practices are single techniques, while others are broad categories that include multiple practices (Ospina, 2007). Additionally, meditation styles from disparate spiritual, religions, and secular traditions have been conflated in the literature and popular press (Awasthi, 2013).

Defining meditation practices is not straightforward. Many of these practices developed within religious, spiritual, and ethical contexts, and they have been appropriated for use in secular or clinical settings. Scientific descriptions of meditation arising in cognitive neuroscience or clinical psychology often omit the context of meditation including beliefs, philosophical positions, rules or guidelines for ethical behavior, cultural background, and other factors considered necessary for effective meditation practice (Dahl et al., 2015; Lutz et al., 2015). Additionally, the procedure for a given meditation practice can differ between traditions or within traditions across different teachers. Furthermore, diverse meditation practices can lead to distinct effects or states of consciousness. A meditative state describes an altered sense of perception, cognitive process, or sense of self that occurs during the course of meditation practice (Cahn and Polich, 2006). It is possible that the same meditation technique could lead to multiple states of consciousness or that different meditation practices could lead to the same meditative states. For example, a meditator attempting to reach a state without discursive thinking might fail to do so during a given meditation session, be successful for only part of a session, or fail to disrupt discursive thinking entirely. Additionally, a meditator could use a variety of techniques to reach this state of nonthinking that might involve distinct cognitive and neural mechanisms. Complicating matters further, meditators can develop altered traits, long-term changes in cognition, or brain dynamics that persist outside of meditation (Lutz et al., 2004). It is possible that meditative states experienced during meditation are mitigated by the altered traits of meditators. Thus, two meditators practicing the same meditation technique might have very different experiences because of their individual traits. It is also possible that a meditator might achieve a state of consciousness without the intentional use of a specific technique because of sustained practice. The interaction between the context of meditation, the specific meditation practices engaged in, and the trait effects of each individual likely play a role in what state of consciousness meditation produces. This logic is true of each of the meditation practices described and outlined below.

In response to the issues outlined above, several classification systems for meditation have been proposed (Nash and Newberg, 2013; Dahl et al., 2015; Lutz et al., 2015; Matko and Sedlmeier, 2019). One classification scheme dichotomizes meditation as focused attention (FA) or open monitoring (OM). FA meditation is used to increase the ability of a meditator to keep their mind fixed on one object (e.g., a visible object, physical sensation, or mental image) for increasing periods of time. This requires the monitoring of external stimuli or thoughts that might take attention away from the target, disengaging from the distraction, and returning attention to the intended target (Fox et al., 2016). Contemplative traditions assert that engaging in a curriculum that incorporates FA meditation reduces mind wandering, increases the stability of attention, and reduces the need to regulate attention through executive skills (Gyatso, 1995). FA meditation is contrasted with OM, which involves turning attention to the present moment and observing all aspects of experiences with an attitude of equanimity (Fox et al., 2016). The FA/OM dichotomy does not fully characterize differences among practices, as illustrated by our descriptions below and in the literature (e.g., Dahl et al., 2015; Lutz et al., 2015).

In the present study, participants engaged in one of six meditation practices: shamatha, vipassana, zazen, dzogchen, tonglen, and deity visualization. All of these practices meet the minimum criteria for contemplative practices outlined by Bond et al. (2009): they involve a defined technique, they include logic relaxation, and they are in a self-induced state. In addition, they meet many of the additional, but not required, features that meditation practices may include, as outlined by Bond et al. (2009). We take a scientific approach to the study of meditation, and we use the FA/OM classification below to illustrate differences among the practices, where possible; however, full explication of the different classification schemes and how the traditions differ from religious or cultural perspectives is beyond the scope of this manuscript. The descriptions below give a general overview of each meditation practice and contain sources from both the scientific and traditional perspectives.

Shamatha is a set of practices used by nearly every school of Buddhism. Shamatha is often translated as “calm abiding.” The literal translation for *shama* is “peace.” *Tha* translates as “abide” or “remain” (Ray, 2004). The term is used to describe a state of mind rather than a practice of meditation. There are various methods designed to achieve calm abiding, with most involving focusing the mind on a single object of attention such as the sensations of the breath or a specific object. This style requires the meditator to develop two faculties: (a) the ability to pay attention to the chosen object and (b) the capacity to notice when the mind has disengaged from the attentional subject unconsciously (Elliott et al., 2014). Generally, shamatha is classified as an FA meditation because most of the time it involves focusing on a single object (Zeidan et al., 2012). However, in some meditation practices, chiefly those originating in Tibet, shamatha can be used in a way that is more similar to OM than FA (Wallace, 1999). In the present study, practitioners of shamatha meditation reported attention to the sensations of the breath as the object of attention, also known as Ānāpānasati (Nanamoli, 2010). If their mind wandered from the task, they brought attention back to the breath.

Vipassana is a Pali word. *Vi* is an adjective suggesting intensity, and “*passana”* translates to “seeing.” Taken together, the word is often translated to “special seeing” (Perdue, 2014). Vipassana meditation was the precursor to the modern mindfulness movement in western countries. It involves purposeful paying attention to the present moment without judgment (Kabat-Zinn, 2003). There are multiple traditions that practice vipassana meditation, all employing a unique style. In the present study, Vipassana meditators practiced in the style of S.N. Goenka. They reported observing the sensations of the body nonjudgmentally by systematically moving attention from head to feet (Hart, 2011; Zeng et al., 2014).

Zazen literally translates to “seated meditation” (Brown and Roshi, 1996). Therefore, as zazen refers to a posture, it is possible that a Zen practitioner could be engaging in either maintaining attention on the breath (FA) or open-awareness (OM) practice during a zazen session. However, in its common usage, zazen refers to the practices of Shikantaza during which a meditator attempts to remain in the present moment (Fischer-Schreiber and Schuhmacher, 1989). In the present study, all zazen participants reported engaging in shikantaza, which means “nothing but sitting” (Fischer-Schreiber and Schuhmacher, 1989). During shikantaza practice, meditators attempted to pay attention to every aspect of experience and view every phenomenon that appears in consciousness as one totality.

Dzogchen translates as the “great perfection,” and it is a collection of meditation techniques practiced by the Nyingma school of Tibetan Buddhism (Van Schaik, 2004). Great perfection refers to a state of recognizing the underlying nature of the mind, the element of experience that is ever-present and unchanging. Dzogchen is often referred to as non-meditation because it can be completed instantaneously by noticing the characteristics of consciousness itself (Van Schaik, 2004). Thus, dzogchen is categorized as a non-dual meditation, falling outside the FA–OM classification structure. During normal waking consciousness or FA/OM meditation, there is a sense self (the subject of subjective experience). Dzogchen practitioners assert that, on both philosophical and experiential levels, there is no difference between subject and object, as both appear inside a field of unbounded awareness. Non-dual awareness, recognized by dzogchen practitioners during non-meditation, is, therefore, awareness itself. To use the often-cited analogy, awareness is like a mirror on which everything appears (Gyatso, 2004). However, in the case of consciousness, there is no separation between awareness and the objects of awareness. In the current study, participants engaged in Trekchö, often translated as “thoroughly cutting through” (Trungpa, 2013). They reported looking at the nature of their minds.

Tonglen is a visualization practice designed to increase one’s capacity for compassion. In Tibetan, *tong* translates to “giving or sending,” and *len* means “receiving or taking” (Drolma, 2019). The practice involves visualizing a specific person, group of people, or geographical area. Upon inhalation, one breathes in his/her suffering while maintaining the aspiration that his/her suffering will decrease. Upon exhalation, one imagines breathing out and sending forth that which might reduce the suffering of those visualized. In the present study, participants who engaged in tonglen practice visualized a person who is suffering and recognized that they wanted this individual to be happy. They then wished the subject to be free of suffering and imagined this suffering turning into a black smoke. The imagined smoke was inhaled by the meditator who visualized its transformation into white light that was exhaled. Upon exhaling, the meditator wished the subject to find happiness (McKnight, 2012).

Deity visualization involves visualizing oneself as a particular being, with particular qualities such as compassion and wisdom that the meditator hopes to embody. The purpose of the practice is to see that these qualities are already intrinsic to every person (Gyatrul, 1969). For example, Tara practitioners visualize themselves as the female Tara deity while reciting a mantra believed to embody the characteristics of compassion and emptiness. The practice is divided into two stages—generation and completion. During the generation stage, meditators imagine themselves as a particular deity while noticing that the image appearing in their minds is like a mirage (Ray, 2002). There are two variations of the completion stage: the path of method and the path of liberation (Kongtrul, 2002). Here, we focus on the path of liberation because that was the method used in the current study. In the path of liberation, meditators attempt to realize the empty nature of reality by experiencing the dissolution of the image they have been visualizing. The goal of the practice is to see how everything is similar to an illusion and that all things have a sense of solidity only by virtue of our having labeled them. During visualization, meditators completed both the perfection stage and the compilation stages, and all visualized the deity Tara (Chodron, 2013).

A goal of many meditation practices is to reduce distraction and mind wandering, and there are neurological correlates suggesting this might be achieved. Research utilizing functional MRI (fMRI) shows distinct forms of meditation activating different brain regions, many of which are implicated in attentional control. For example, compared with OM, FA meditation is correlated with increased activity and connectivity in the anterior cingulate cortex (Lazar et al., 2000; Manna et al., 2010). Both FA and OM result in increased activity in the insula during introspection compared with controls (Farb et al., 2007). Researchers have also noted reductions in default mode network (DMN) activity during both FA and OM meditation, with less activation in the ventral medial prefrontal cortex, precuneus, medial temporal lobe, and posterior cingulate gyrus (Hölzel et al., 2007; Brewer et al., 2011; Simon and Engström, 2015; Lee et al., 2018). Thus, there is a growing body of literature to suggest that different styles of meditation are associated with distinct patterns of neuronal activation and connectivity.

Many studies investigating the neural correlates of meditation use EEG. EEG is a technique that monitors the activity of neurons with an array of highly conductive sensors placed over the scalp. These sensors measure the voltage produced by neurons (Henry, 2006). EEG oscillatory activity occurs when a large number of postsynaptic potentials occur simultaneously (Kirschstein and Köhling, 2009). A growing body of evidence suggests oscillations, quantified as power spectra, are important for coordinating information exchange between brain regions and promoting neural plasticity (Engel et al., 2001; Varela et al., 2001; Buzsáki and Draguhn, 2004; Fries, 2005). Oscillatory activity changes based on states of consciousness (Davidson, 1976; Dietrich, 2003). During meditative states achieved by highly experienced meditators, changes in EEG oscillatory signatures have been reported (Lutz et al., 2004). While the neuroelectric correlates of meditation have not been fully explored, earlier research suggests that, in general, meditative states produce increases in the power of theta and alpha (for reviews, see Delmonte, 1984; Andresen, 2000; Dietrich, 2003; Cahn and Polich, 2006; Fell et al., 2010; Lomas et al., 2015).

Together, the previous findings on the neural correlates of meditative states from EEG and fMRI studies provide converging evidence that some meditative states are significantly different from other states of consciousness at the level of the brain (Davidson, 1976; Dunn et al., 1999; Dietrich, 2003; Lutz et al., 2007). Additionally, there is a growing body of evidence suggesting that different styles have unique effects on the EEG signal (Dunn et al., 1999; Lutz et al., 2008; Travis and Shear, 2010; Tomasino et al., 2013; Fox et al., 2014, 2016; Lomas et al., 2015). Dunn et al. (1999) examined the effects of concentration meditation with the breath as the object (shamatha) compared with a resting state. During meditation, they found a decrease in average theta and increases in posterior alpha and posterior beta. The researchers found significant differences between the EEG signal produced by concentration meditation and mindfulness meditation. However, this study used student volunteers who, after training, had fewer than 100 h of lifetime practice. Saggar et al. (2012), using a longitudinal design of 3 months with waitlist controls, found that observing the sensations of the breadth in shamatha meditation resulted in reduced alpha and beta band power.

The style of vipassana as taught by S.N. Goenka has also received some attention from neuroscience researchers. A group of high-level meditators showed no difference in theta, alpha, or beta bands but reported increases in occipital gamma (Cahn et al., 2010). In contrast, vipassana meditators showed increases in the delta, theta, and alpha bands (Kakumanu et al., 2018).

Zen meditation was the focus of early meditation research. Kasamatsu and Hirai (1966) conducted a large study with Zen priests. They found increased alpha amplitude during meditation compared with a rest control condition. Murata et al. (1994) reported increased frontal midline theta in advanced meditators engaging in zazen, and a more recent study found decreased EEG theta and beta power in the frontal region during Zen meditation (Hauswald et al., 2015).

To our knowledge, the electrophysiological correlates of dzogchen and visualization have each been explored in only one study. Schoenberg et al. (2018) reported increased gamma-band current density within brain regions associated with self-referential processing such as the anterior cingulate cortex, precuneus, and superior parietal lobule during essence-of-mind practice. An increase in beta-band activity in the insula was also reported. Amihai and Kozhevnikov (2014) demonstrated reduced beta power during visualization compared with a rest condition. To date, there has been no research on the neurological correlates of tonglen.

Another way to explore neurological signatures of different tasks is the use of nonlinear data analysis tools. New research in brain dynamics suggests that the modulations in the variability of neural signals are important for healthy cognition (Armbruster-Genç et al., 2016). In other words “complexity lies between order and disorder” (Erra et al., 2016). Early evidence suggests that, counterintuitively, variability is necessary for stable cognitive and behavioral outputs of the brains (Pinneo, 1966; Pakhomov and Sudin, 2013).

A popular subset of these methods centers on entropy, “a dimensionless quantity that is used for measuring uncertainty about the state of a system but it can also imply physical qualities, where high entropy is synonymous with high disorder” (Carhart-Harris et al., 2014). Measures of entropy are becoming more common in neuroscience including research with altered states of consciousness such as sleep, anesthesia, and psychedelic states (Cavanna et al., 2018).

The use of entropy in the current study was informed by Robin Carhart-Harris’s entropic brain theory based on neuroimaging work with psychedelic drugs (Carhart-Harris et al., 2014; Carhart-Harris, 2018). Carhart-Harris argues that measures of brain entropy are useful because they allow researchers to make qualitative observations based on the quantitative measure of randomness as measured by neuroimaging techniques. For example, a low-entropy brain signal is reported when the content of consciousness is reduced [non-rapid eye movement (NREM) sleep, anesthesia, or coma], and high-entropy brain signals have been reported as phenomenologically rich (psychedelic drugs). Lempel–Ziv complexity (LZc) is one approximation of entropy (Lempel and Ziv, 1976). LZc provides a measure of entropy by “counting the number of distinct patterns of activity in the data. It can be thought of as being proportional to the size of a computer file containing the data, after applying a compression algorithm” (Schartner et al., 2015). We chose this method because it has been used research on altered states of consciousness such as awake, general anesthesia, psychedelics, and mental health disorders including depression, anxiety, and schizophrenia (Radhakrishnan and Gangadhar, 1998; Thomasson and Pezard, 1999; Gómez et al., 2006; Fernández et al., 2011, 2013; Méndez et al., 2012; Bachmann et al., 2015; Hudetz et al., 2016; Schartner et al., 2017a; Timmermann et al., 2019).

In the present study, we compared EEG of highly skilled meditators while meditating and engaging in a mind-wandering task. We used a mind-wandering task as a baseline control because expert meditators have trained their minds to remain in a meditative state at all times, making it difficult to achieve a non-meditative baseline without an active task (Lutz et al., 2004). Using a 16-channel system, we captured delta, theta, alpha, beta1, beta2, and beta3 across the two conditions within the same participants during the initial 600 s of meditation. We calculated entropy during the meditation and the mind-wandering tasks using the LZc approximation (Lempel and Ziv, 1976). We also compared power spectra at six different frequency bands across the two tasks. For the entropy measure, we predicted lower values during meditation compared with mind wandering, similar to findings with other altered states. Power spectrum differences, both global and local, are a standard measure of EEG activity; however, which bands are activated during meditation varies across studies; thus, we have no *a priori* predictions for these measures (Cahn and Polich, 2006; Lomas et al., 2015; Schoenberg et al., 2018). Finally, exploratory analyses investigated how the six practices of meditation compared with each other neurophysiologically.

## Materials and Methods

### Participants

Meditative communities were contacted in India, Nepal, and the United States to assess interest in participating in the study. Meditation instructors within the community selected participants who had a high level of experience. Each community provided a space to conduct the recordings, usually in the area designated for meditation practice. Participants were not compensated for their time. The study was approved by the Colby College Institutional Review Board, and participants provided written informed consent. Forty-two participants were recruited. Fourteen participants were excluded due to a history of traumatic brain injury, unusable recordings, or lack of meditation experience or because they described their meditation style as phenomenologically different from that of other participants. Participants studied at least one of six practices—zazen, dzogchen, shamatha, visualization, vipassana, and tonglen. Participants in each meditative group had undergone instruction in the same school of meditation. In some cases, participants completed multiple recording sessions for different practices. For these participants, we analyzed only their first meditation recording. The resulting sample size was 28 participants (seven shamatha, six zazen, six vipassana, five dzogchen, three visualization, and one tonglen).

Participants had a mean age of 52.32 (SD = 15.74; 14 females), an average of 21,934.64 h (SD = 20,186.46) of lifetime practice with 7,308.50 (SD = 10,177.70) of those hours completed while on retreat. A day of retreat equaled 6 h or more of practice. All but two were right-handed. See Supplementary Materials for more demographic information.

### Meditation Practices

All of the meditation methods involved intentional monitoring of emotion regulation or attention, but the focus during the meditations differed. Each participant participated in a semi-structured interview to determine the phenomenological character of their meditation style. This interview was used to determine if individuals using a shared label for their meditation practice were engaging in a similar cognitive process during meditation. The interview was guided by a questionnaire adapted from Lutz et al. (2007) and is included in the Supplementary Materials.

### Procedure

A verbal description of the study rationale and procedures was presented followed by a period for questions. After written informed consent was obtained, a questionnaire assessing meditative experience, biographical information, and screening for past neurological abnormalities was administered. Then, a semi-structured interview determined if the phenomenological character of each meditator’s unique style was similar to that of the group.

EEG activity was recorded according to the International 10–20 System with 16 channel Ultracortex Mark IV (OpenBCI, New York, NY, United States), sampled at 250 Hz, referenced with linked earlobe sensors. A digital notch filter was applied to the data at 50 Hz for data collected in India and Nepal and 60 Hz for data in the United States to remove alternating current line noise. Sixteen electrodes were placed (Fp1, Fp2, F3, F4, F7, F8, C3, C4, T3, T4, T5, T6, P3, P4, O1, and O2) on the scalp. Impedances levels were less than 10 kΩ before recording to ensure electrodes were in good contact with the scalp.

Participants sat in the same posture for both meditation and mind-wandering tasks. We first collected an initial EEG baseline consisting of one 80-s block. While sitting in their usual meditation posture, participants were instructed not to engage in any meditation technique and instead to think about their day starting when they woke up. This condition emulated a mind-wandering state to be contrasted with the purposeful, attentional engagement in the present moment while in a meditative state (Christoff et al., 2009; Smallwood and Schooler, 2006).

Following the baseline, participants were instructed to begin meditation, which was recorded for 600 s from the beginning of their meditation session. We did not ask what state they achieved during this time. After recordings were completed, participants were asked if the recording equipment had interfered with their meditation practice. No participants indicated that the device had interfered with their meditation.

### Electrophysiological Analyses

EEG preprocessing was conducted in EEGLAB (Delorme and Makeig, 2004) using a custom script that was implemented in MATLAB (Math Works Inc., Natick, MA, United States). The time series of raw data were visually inspected for artifacts. Periods with non-local artifacts involving many electrodes were identified visually and removed. A high pass filter at 0.5 Hz and a low pass filter at 80 Hz were applied using finite impulse response (FIR) filters (Rabiner and Gold, 1975). Eye movement artifacts were removed using independent component analysis (ICA; Jung et al., 2000).

To compare changes in EEG activity between meditation and mind-wandering conditions, we first computed the power spectral density during each condition for each channel using the fast Fourier transform (FFT) in EEGLAB. In EEG power spectra, like many physical signals, power scales with frequency; i.e., lower frequencies are present in higher power than high frequencies. The power law scaling relationship for EEG signals is represented by S(f) = 1/f, where S(f) is the power spectral density and f is the frequency from 0 to ∞. It is a parameter that can be unique to the individual and can change with age (Voytek et al., 2015) and is influenced by external sources of noise in the recording (Keshner, 1982). This so-called 1/f noise can distort measurements of EEG power, especially at the lower frequencies known to be influenced by meditative state (Demanuele et al., 2007). For the present experiment, the varied ages of participants and especially the varied conditions encountered recording at the field sites led us to remove this source of variability from our measures. To normalize the power spectra, we independently fit the power spectrum for the meditation and mind-wandering conditions for each participant with a polynomial of the form 1/f, and we subtracted this trend from the raw power spectrum.

### Statistical Plan

To compare measures of entropy and normalized power spectra in the meditation and mind-wandering conditions, we used SPSS v. 24 to conduct repeated-measures ANOVAs. Our measure of entropy was based on LZc. We calculated the LZc in python with a script provided by Schartner et al. (2015). For the entropy analyses, a post-hoc statistical power analysis was conducted for our repeated-measures ANOVAs with two groups and two measures (alpha = 0.05 and power = 0.95). For a moderate effect size (Cohen’s f = 0.44), a sample size of *N* = 14 resulted in a 0.86 actual power (using GPower 3.1.9). Cohen’s f was determined from our obtained partial *η*^2^ (0.16; see the section “Entropy”) using the following conversion: Cohen’s f = square root of [*η*^2^/(1 - *η*^2^)] (with one factor partial *η*^2^ = *η*^2^). For the analyses of power spectra, the largest obtained partial *η*^2^ = 0.10 (Cohen’s f = 0.33; see Table 1). Power analysis using the same parameters as for the entropy analysis revealed that a sample size of *N* = 22 resulted in 0.84 actual power.

**TABLE 1:** Mean (and standard deviation) power spectra as a function of frequency band and state.

Band	Mind wandering	Meditation	*p*^*a*^	Partial *η*^2^
Delta	0.58 (0.47)	0.66 (0.34)	0.430	0.02
Theta	−1.27 (1.13)	−1.51 (0.87)	0.197	0.06
Alpha	0.95 (1.22)	1.25 (1.00)	0.094	0.10
Beta1	−0.30 (0.96)	0.50 (0.80)	0.095	0.10
Beta2	0.07 (0.29)	0.03 (0.26)	0.611	0.01
Beta3	−0.15 (0.23)	−0.12 (0.18)	0.482	0.02

*^a^p Values represent tests for differences between the two state conditions.*

## Results

### Entropy

To test for differences in global entropy during meditation and mind wandering as measured by LZc, we conducted a one-way ANOVA with state (meditation, mind wandering) as a within factor. The analyses revealed a significant main effect for state (*F* (1, 27) = 5.24, *p* = 0.030, partial *η*^2^ = 0.16). LZc scores were lower during meditation (*M* = 0.84, SD = 0.11) than during mind wandering (*M* = 0.88, SD = 0.10). Neither entropy measures were significantly correlated with age: *r*s = 0.23 and 0.35, *p*s = 0.240 and 0.068.

### Power Spectra by Frequency Band

We averaged the power spectra across all channels and created the following frequency bands: delta (1–4 Hz), theta (4–8 Hz), alpha (8–12.5 Hz), beta1 (12.5–16 Hz), beta2 (16.5–20 Hz), and beta3 (20.5–28 Hz). One-way ANOVAs for each band compared power spectra across meditation and mind-wandering sessions. All analyses showed no significant differences (mean, standard deviation, *p*-value, and effect size in Table 1). We found small effect sizes for delta, theta, beta2, and beta3; and we found medium effect sizes for alpha and beta1. The reason for the lack of significant differences could be due to the variability introduced by the fact that the participants engaged in different meditative practices. We explore the possibility that different practices show different neurophysiological profiles in the next section.

### Exploratory Analyses by Practice

**FIGURE 1:**
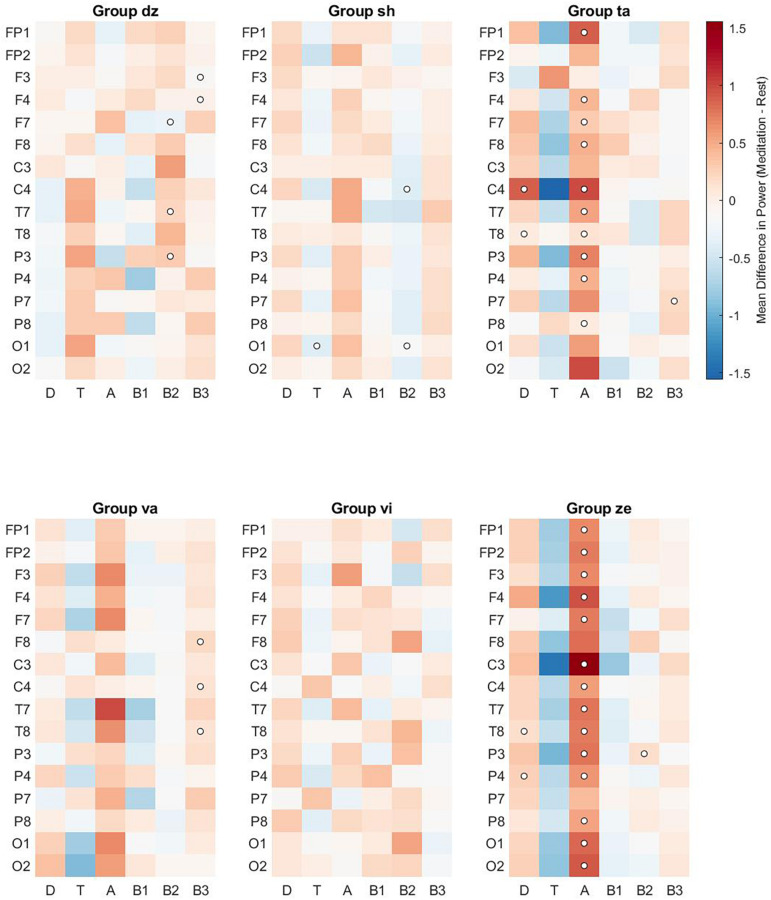
Mean differences in power from meditation and mind-wandering conditions for each of the six meditation groups. Fields with a white circle were significant at the 0.05 level after false discovery rate (FDR) correction for recording sites, frequency bands, and conditions. Sh, shamatha; Va, vipassana; Ze, zazen; Dz, dzogchen; Ta, tonglen; and Vi, visualization.

Because we did not have enough participants in each practice for sufficient statistical power, we highlight descriptive differences across practices. First, we looked at differences between the practices at each frequency band (Figure 1). Significant differences in power between conditions at each site were determined by a two-tailed *t*-test implemented in EEGLAB, corrected for multiple comparisons by the false discovery rate (FDR) procedure (Benjamini and Yekutieli, 2001; *p* < 0.05). Participants who engaged in tonglen and zazen showed the largest significant increases in alpha power across the majority of electrodes in all regions.

**FIGURE 2:**
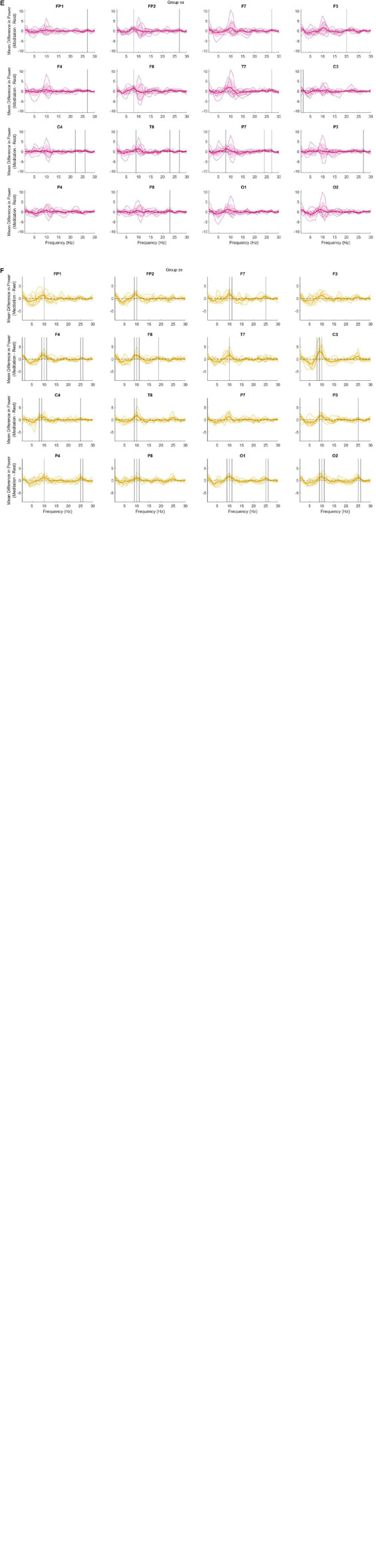
The difference (meditation minus mind wandering) in power across frequency bands. Thin colored lines represent a single participant, and the bold-colored line indicates the mean. The shaded regions indicate 1 standard deviation from the mean. Vertical black lines indicate a significant difference between conditions at the 0.05 level with bootstrap statistics and with false discovery rate (FDR) correction. **(A)** dzogchen; **(B)** shamatha; **(C)** visualization; **(D)** tonglen; **(E)** vipassana; and **(F)** zazen.

The preliminary analysis also shows notable differences between groups in the power spectrum for some meditation practices that are not revealed in Figure 1. Figure 2 demonstrates these differences using data not separated into the frequency bands. Of note is the large variability present in the dzogchen (Figure 2A), shamatha (Figure 2B), and visualization groups (Figure 2C). In contrast, tonglen (Figure 2D), vipassana (Figure 2E), and zazen (Figure 2F) meditators had qualitatively lower levels of within-group variability. The findings shown in Figure 2 may be of value to future researchers studying one or more of these meditation styles.

## Discussion

The purpose of this study was to examine the neurophysiological responses by highly skilled meditators while meditating and while engaging in a mind-wandering task. We used two measures, entropy and power spectra, at six different frequency bands. As predicted, we found a difference in entropy, with lower amounts of entropy during meditation compared with mind wandering. This finding contrasts with that of Vivot et al. (2020), who found increased entropy of band-specific oscillations during three styles of meditation—Himalayan Yoga (FA), Vipassana (OM), and Isha Shoonya Yoga (non-dual). One reason for the difference in findings is that Vivot et al. (2020) calculated entropy based on specific frequency bands rather across all bands, as we did here. Reduced complexity during meditation has been reported in one study investigating Sahaja Yoga meditation, a style characterized by lack of thoughts (Aftanas and Golocheikine, 2002).

While complexity analysis is relatively new in the field of contemplative neuroscience, there is a wealth of research on entropy during other altered states of consciousness. For example, entropy decreases during general anesthesia (Bruhn et al., 2000; Zhang et al., 2001; Schartner et al., 2015), vegetative state (Burioka et al., 2005; Sarà and Pistoia, 2010; Gosseries et al., 2011; Wu et al., 2011, Schartner et al., 2017b), and sleep in both humans and animals (Hudetz et al., 2016). In addition, entropy increases with serotonergic psychedelics such as *N*,*N*-dimethyltryptamine (Timmermann et al., 2019), lysergic acid diethylamide, psilocybin, and ketamine (Tagliazucchi et al., 2014; Schartner et al., 2017a). Finally, entropy is decreased in certain neurological conditions such as epilepsy (Radhakrishnan and Gangadhar, 1998), Alzheimer’s disease (Gómez et al., 2006), attention deficit/hyperactivity disorder (ADHD; Fernández et al., 2009, and depression (Pezard et al., 1996; Li et al., 2008; Akar et al., 2015; Bachmann et al., 2015). The level of complexity normalizes with pharmacological treatment (Méndez et al., 2012), repetitive transcranial magnetic stimulation (Lebiecka et al., 2018), and electroconvulsive therapy (Thomasson and Pezard, 1999). Measures of complexity are also abnormally high in patients with schizophrenia (Li et al., 2008; Fernández et al., 2011; Fernández et al., 2013).

It is believed that low complexity in the brain reflects a smaller number of possible configurations, or conscious awareness with less experienced content (Schartner et al., 2017a; Vivot et al., 2020). Similarly, high-entropy states may be rich in phenomenological content (Tagliazucchi et al., 2014; Carhart-Harris, 2018; Cavanna et al., 2018; Vivot et al., 2020). We found significantly reduced complexity during meditation. Most practices included in this study explicitly involve the reduction in the number of sensory modalities in focus. Shamatha, tonglen, vipassana, and visualization all involve focusing on one or more sensory modality at the exclusion of others. Attention is directed to specific senses in shamatha (sensation of breath), vipassana (sensations of the full body), tonglen (imagined imagery and emotion), and visualization (imagined imagery). Dzogchen participants focused on the substrate of awareness without explicitly excluding any appearances in awareness. Our results suggest that limiting attention to sensory modalities is associated with decreased levels of complexity in the EEG signal.

In contrast to entropy, we found no difference in power spectra between meditation and mind wandering. The findings are mixed regarding whether differences should be found in each band as a function of meditating or not (Delmonte, 1984; Andresen, 2000; Dietrich, 2003; Cahn and Polich, 2006; Fell et al., 2010; Lomas et al., 2015). For example, delta effects are rarely reported in the meditation literature (Cahn et al., 2010), and our small effect size is consistent with this finding. One study reported increased 2- to 4-Hz power in response to oddball stimuli in meditators compared with controls (Cahn et al., 2013). Delta has been studied extensively during sleep and has been linked to a number of processes including neuronal plasticity (Steriade and Timofeev, 2003). Delta is involved in a wide range of cognitive processes, many of which involve motivation and the brain reward system (Knyazev, 2007).

Theta oscillations have been implicated in learning tasks during event-related potential (ERP) and continuous recording conditions. Increases in theta activity have been found in working memory (Raghavachari et al., 2001; Jacobs et al., 2006; Hsieh et al., 2011), recall (Grunwald et al., 1999; Sederberg et al., 2003), and spatial memory and navigation (Kahana et al., 1999; Araújo et al., 2002; Caplan et al., 2003; Watrous et al., 2011). Decreased theta is associated with difficulty in episodic retrieval (Addante et al., 2011). Increases in theta power have been reported in the meditation literature for both FA and OM (Aftanas and Golocheikine, 2001, 2002; Baijal and Srinivasan, 2010; Pasquini et al., 2015; Banquet, 1973; Jacobs and Lubar, 1989; Pan et al., 1994), and increased theta has become a key feature of meditation (Lagopoulos et al., 2009; Fell et al., 2010; Josipovic, 2010). In some cases, increases in theta power were correlated with meditation experience (Lee et al., 2018). In contrast to this previous work, we found no effect of meditation on theta.

There is no agreed-upon explanation of the function of alpha (Bazanova and Vernon, 2014); however, increased alpha in frontal areas has been found in mindfulness meditation (Takahashi et al., 2005) and in posterior regions (Lagopoulos et al., 2009). Advanced meditators in the Satyananda Yoga practice had higher levels of alpha than had novice practitioners (Thomas et al., 2014). Similarly, zazen and vipassana practitioners showed increases in alpha during meditation (Kasamatsu and Hirai, 1966; Braboszcz et al., 2017). We too found a moderate effect size for alpha across the meditation and mind-wandering conditions, albeit the overall difference was not significant.

Beta oscillations have been linked to the sensorimotor cortex, specifically when sensory motor activity is actively being maintained (Engel et al., 2001; Brovelli et al., 2004). We chose to separate the beta band into three distinct groups after Hinterberger et al. (2014). Increased beta1 has been linked to meditative states. Dunn et al. (1999) found increased beta1 in 10 of 19 recording sites across a number of brain regions when concentration meditation was compared with a relaxation baseline. Moreover, more mean beta1 activity was found during mindfulness meditation in 14 of 19 recording locations as compared with the same relaxation condition (Dunn et al., 1999). Other studies found increased beta (defined as 13–30 Hz) during mindfulness meditation compared with a control state (Ahani et al., 2014; Schoenberg et al., 2018). In contrast, Amihai and Kozhevnikov (2014) found decreased beta2 power during the compilation stage of a visualization meditation, and a third study found both an increase in beta2 (6 of the 19 sites) and a decrease (4 of the 19 sites) when comparing concentration meditation with a relaxation baseline. Beta3 activity was suppressed during shamatha meditation with breath as the object of focus (Saggar et al., 2012). Studies with vipassana meditation found no change in beta-band activity (Cahn et al., 2010). Our effect sizes show an effect for beta1 but not for beta2 and beta3, highlighting the need for more research to understand how beta is affected by meditation.

Given that power spectra tap different processes in different frequency bands, it is no wonder we did not find differences in power spectra in our sample. Our participants engaged in one of six practices, and the variability in neurological response as a function of practice may have precluded our ability to see any overarching trends. In light of these findings, we engaged in exploratory analyses to see potential areas of difference, which may guide future research. These analyses suggest that different meditation practices may have different neurological signatures, particularly in terms of power spectrum bands.

Consistent with Lutz et al. (2015), we argue that providing succinct descriptions of practices under study should always be included in investigations. Most meditation research lacks clear descriptions of the practice being studied (Ospina, 2007; Davidson and Kaszniak, 2015). For example, zazen meditation is often classified as OM, ignoring the multiplicity of psychological practices that can be used during zazen (Faber et al., 2015). Initial zazen training includes engaging in a concentrative practice focusing on the breath (FA), while later training incorporates open-awareness practice (OM) during seated meditation. Without an accurate description of the precise practices requested of participants, it is possible that when asked to perform zazen, participants in the same study are engaging in different mental exercises. The same logic is true of other practices such as shamatha, vipassana, and visualization. In the present study, multiple participants appeared to share practices based on survey answers, but first-person descriptions revealed very distinct meditation methodologies. For example, many participants listed “Burmese vipassana” as their meditation style on the questionnaire. However, during the interview about their practice, it was clear that this technique could be implemented using one of two different meditation practices. The first was in the lineage of Mahasi Sayadaw, which involves noting ongoing changes in all sensory domains. The second was in the lineage of Sayagyi U Ba Khin popularized by S.N. Goenka, and meditators focused exclusively on body sensations. Thus, if accurate phenomenological descriptions are not included along with experimental findings, future researchers cannot confidently make comparisons between the results of separate studies that focus on the same style of meditation.

### Strengths

The within-participant comparison of EEG during meditation and mind wandering across six practices is a strength of this investigation. Because of the heterogeneity of meditation practices, we identified that entropy is not practice specific. In other words, during meditation, regardless of practice, participants showed lower entropy than in the mind-wandering task. Thus, entropy is affected by the practice of meditation in general, and it is not tied to any specific activity, such as FA or OM. Power spectra, in contrast, are likely affected by the meditation practice, although our evidence is indirect and based on null findings. The heterogeneity of practices may have precluded our ability to identify any differences between meditation and mind wandering across six frequency bands.

A second strength is our use of the mind-wandering task. Participants were asked to “think about your day since the moment you woke up this morning” to achieve baseline conditions with less heterogeneity than the standard rest condition and to prevent highly skilled meditators from automatically engaging in meditation. Mind wandering is characterized by self-directed thoughts of the past and future (Smallwood et al., 2009; Smallwood and Schooler, 2015; Christoff et al., 2009; Stawarczyk et al., 2011). Moreover, meditation is used as a tool to decrease mind wandering and increase time in the present moment. Some studies have suggested that meditation alters the DMN (Pagnoni et al., 2008; Tei et al., 2009; Brewer et al., 2011; Farb et al., 2007), which is implicated in mind wandering. At the same time, any control task is open to criticism when working with experienced meditators because meditation has lasting effects outside of the actual session, a topic we address below.

### Limitations

One limitation of the study is sample sizes for the six practices. Testing participants in three different countries where they typically mediated (e.g., a monastery) still resulted in small number of participants per tradition. Thus, we were unable to make cross-tradition comparisons beyond the exploratory findings reported in the results section. We also were constrained in our analyses by using a low-density (16-channel) electrode array. For example, higher-density (256-channel) arrays allow researchers to make inferences about source location of EEG signals. Another limitation is the fact that we did not ask participants whether they reached the targeted state during the 600 s of recording. Some studies collect EEG data only once the participant has reached a meditative state. Our focus was on the beginning phases of meditation, and it is possible that some participants, but not all, reached their meditative state within that time interval.

A challenge for this study and for the field generally may be the fact that the majority of meditation research has focused on power spectra. There is wide variation in the power spectrum profile in the normal population and over an individual’s lifetime (Haegens et al., 2014; Voytek et al., 2015; Hashemi et al., 2016; Parameshwaran and Thiagarajan, 2017). Also, there is no agreement on the range for different frequency bands. For example, frequencies considered for delta could begin between 0 and 2 Hz and end between 3.5 and 6 Hz. In addition, some studies do not report findings for all frequency bands, potentially leading to bias toward positive results (Newson and Thiagarajan, 2019). Relatedly, power spectrum analysis is limited due to the lack of specificity to underlying neuronal processes. For example, using frequency band analysis for differentiating psychiatric disorders is ineffective because there is too much overlap between disorders (Newson and Thiagarajan, 2019). Power spectrum analysis of different meditation styles is similarly limited. The power spectrum of the EEG signal alone is similar to describing a digital image using the color spectrum. This would give a general idea of the content (more blue in the image could mean the photo is of the sky or ocean). However, when studies report the global power spectrum, the EEG signal loses spatial and temporal dimensions. Similarly, the power spectrum lacks temporal information, which is one of the main advantages of EEG recording compared with other neuroimaging techniques (Newson and Thiagarajan, 2019).

Thus, more sophisticated data analytical techniques are needed to study the EEG correlates of meditation, such as functional connectivity measures and analyses that explore the temporal component of the EEG signal. In addition, data repositories and processing pipelines should be created and implemented to allow for standardization in the field and to allow for comparisons of results across researchers. Additionally, the potential difficulties in differentiating meditation practices based on the power spectrum raise questions about the efficacy of EEG-assisted meditation. We did not find many differences using a 16-channel system. Many devices for EEG-assisted meditation have less than four recording locations. We suggest that the efficacy of these devices be assessed.

Finally, the study of meditation presents unique methodological challenges. Many meditation styles are purported to have lasting psychological effects outside of a meditation session. The distinction between neurological or psychological changes that occur during meditation and changes that persist over time has been called state and trait effects, respectively (Cahn and Polich. 2006). Some studies show trait differences between normative resting EEG spectra and the baseline spectral profile of meditators (Lutz et al., 2004). Thus, a limitation of the present study is the absence of a non-meditator control group. This limits our ability to study the trait effects of meditation. However, finding a matched control group for highly skilled meditators is very difficult. People who accrue tens of thousands of hours of meditation generally have different environmental factors including diet, social interaction, and other psychological factors such as lack of stress than potential non-meditator control participants (Davidson, 2010). Because of these limiting factors, a longitudinal study comparing the electrophysiological and psychological correlates of meditation practices is indispensable if we hope to increase our understanding of both state and trait effects.

## Conclusion

By comparing EEG activity in the same person while engaging one of six meditation practices and while engaging in a mind-wandering task, we found that meditation reduces neural complexity regardless of meditation practice. In contrast, it is likely that different practices affect power spectra in different ways. Meditation describes a wide range of practices each with a unique goal. Whether these different practices have distinct effects in the brain remains an open question.

## Data Availability Statement

The original contributions presented in the study are publicly available. This data can found here: https://github.com/JacobHYoung/Contrasting-EEG-Derived-Entropy-and-Neural-Oscillations-During-Six-Meditation-Practices.git.

## Ethics Statement

The studies involving human participants were reviewed and approved by Colby College Institutional Review Board. The patients/participants provided their written informed consent to participate in this study.

## Author Contributions

JY and MA designed the study, performed the statistical analysis, and revised the work in light of reviewers’ comments. JY collected data, performed data preprocessing, and performed analysis for extracting LZc scores. JY and JM performed analysis of oscillatory activity. JY wrote the first draft of the manuscript and MA provided comments and editing assistance. All authors contributed to the clarity and accuracy of the manuscript.

## Conflict of Interest

The authors declare that the research was conducted in the absence of any commercial or financial relationships that could be construed as a potential conflict of interest.
