# Supplementary material for: Contrasting Electroencephalography-Derived Entropy and Neural Oscillations With Highly Skilled Meditators
Source: Front Hum Neurosci. 2021 Apr 30;15:628417. doi: 10.3389/fnhum.2021.628417 (PMC8119624; doi:10.3389/fnhum.2021.628417)
Supplement: Supplementary file 1 [file Data_Sheet_1.PDF]

Supplementary Material

1. Participant Questionnaire

ID#:

Demographic:

Date of birth	
Gender	
Country of Birth	
Height	
Weight	
Occupation	
Education	
Handedness	

Health:

1. Had Seizures Before? (Yes / No)
2. Ever had brain surgery? (Yes / No)
3. History of brain tumor? (Yes / No)
4. History of central nervous system infections? (Yes / No)
5. History of head trauma? (Yes / No)

a. Date of Trauma:
b. Loss of Consciousness? (Yes / No)

Meditative Experience:

Year Started Meditating	
Meditation Days Per Year	
Average Meditation Hours Per Day	
**Total**	

Retreat Experience:

Retreat Duration (Days)	Number of retreats	Hours Per Day
**Total**		

Meditation Styles:

Technique practiced	Percentage of lifetime practice

Total hours:

2. Questionnaire to Differentiate Mediation Styles

1. Concerning stability and clarity:
    a. In view of the practitioner's level, should the meditation favor stability, clarity, or a balance?

    b. What are the indications that stability needs adjustment?

    c. What are the indications that clarity needs adjustment?

2. Concerning intentional modality:
    a. If the meditation includes an object, then:
        i. Is there one object or many objects in the meditation?

        ii. For each object, is the object dynamic or static?

        iii. If the object includes or consists of a visual form, a sound, or a sensation, then is the object perceived through the senses, or is it imagined in the mind through visualization or another technique?

    b. If the meditation does not include an object, then does one direct one's attention to something else?

3. Concerning meditative techniques:

a. Is the practice done with the eyes opened or closed?

b. Does the practice employ any discursive strategies, such as recitations, memorized descriptions or arguments that one reviews?

c. Does the practice use breath manipulation?

d. Does the meditation involve focusing on different parts of the body by means of a visualization or some other technique?

e. Does the practice require a specific posture or set of physical exercises?

4. Concerning expected effects during meditation:
    a. Is the meditation expected to produce any physical sensations or mental events, either constantly or intermittently?

    b. Does one expect the meditation to produce subjectively noticeable alterations in cognition, either constantly or intermittently?
        i. One example would be the impression that one's perceptions seem to be like the appearances in a dream.

    c.
    d. Is the meditation expected to cause any emotions, either constantly or intermittently?

5. Concerning expected effects after meditation:
    a. Does one expect the meditation to alter one's cognitions? One example would be the impression that one's perceptions are more vivid.

    b. Does one expect the meditation to alter one's behavior? One example would be a tendency to sleep less.

    c. Does one expect the meditation to alter one's emotions? One example would be the tendency to recover more quickly from emotional disturbances.
    d.
6. Are there any further distinguishing features of the meditation bedsides those mentioned above?
